# POPI (Pediatrics: Omission of Prescriptions and Inappropriate Prescriptions): Development of a Tool to Identify Inappropriate Prescribing

**DOI:** 10.1371/journal.pone.0101171

**Published:** 2014-06-30

**Authors:** Sonia Prot-Labarthe, Thomas Weil, François Angoulvant, Rym Boulkedid, Corinne Alberti, Olivier Bourdon

**Affiliations:** 1 Pharmacie, AP-HP Hôpital Robert-Debré, Paris, France; 2 Pharmacie Clinique, Université Paris Descartes, Paris, France; 3 Service d’Accueil des Urgences, AP-HP Hôpital Robert-Debré, Paris, France; 4 Unité d’Epidémiologie Clinique, AP-HP Hôpital Robert Debré, Paris, France; 5 Inserm U 1123 et CIC 1426, Paris, France; 6 Sorbonne Paris Cité UMRS 1123, Université Paris Diderot, Paris, France; 7 Laboratoire Educations et Pratiques de Santé, Université Paris XIII, Bobigny, France; Nottingham University, United Kingdom

## Abstract

**Introduction:**

Rational prescribing for children is an issue for all countries and has been inadequately studied. Inappropriate prescriptions, including drug omissions, are one of the main causes of medication errors in this population. Our aim is to develop a screening tool to identify omissions and inappropriate prescriptions in pediatrics based on French and international guidelines.

**Methods:**

A selection of diseases was included in the tool using data from social security and hospital statistics. A literature review was done to obtain criteria which could be included in the tool called POPI. A 2-round-Delphi consensus technique was used to establish the content validity of POPI; panelists were asked to rate their level of agreement with each proposition on a 9-point Likert scale and add suggestions if necessary.

**Results:**

108 explicit criteria (80 inappropriate prescriptions and 28 omissions) were obtained and submitted to a 16-member expert panel (8 pharmacists, 8 pediatricians hospital-based −50%- or working in community −50%-). Criteria were categorized according to the main physiological systems (gastroenterology, respiratory infections, pain, neurology, dermatology and miscellaneous). Each criterion was accompanied by a concise explanation as to why the practice is potentially inappropriate in pediatrics (including references). Two round of Delphi process were completed via an online questionnaire. 104 out of the 108 criteria submitted to experts were selected after 2 Delphi rounds (79 inappropriate prescriptions and 25 omissions).

**Discussion Conclusion:**

POPI is the first screening-tool develop to detect inappropriate prescriptions and omissions in pediatrics based on explicit criteria. Inter-user reliability study is necessary before using the tool, and prospective study to assess the effectiveness of POPI is also necessary.

## Introduction

Rational use of medicines refers to the correct, proper and appropriate use of medicines. The WHO estimates that over 50% of medications are prescribed, dispensed or sold inappropriately and that more than 50% of all countries do not implement basic policies to promote rational use of medicines [1]. In developing countries, less than 40% of patients in the public sector and 30% in the private sector are treated according to clinical guidelines [1]. The use of medication in pediatrics should be based on established recommendations from well-conducted clinical trials, however in the absence of such trials, recommendations are often based on clinical experience. Rational prescribing for children is an issue for all countries and has been inadequately studied [2], [3].

The Medical Subject Headings (MeSH) tool is a thesaurus integrated into the PubMed search engine that allows access to the MEDLINE database. In 2011, it introduced the term ‘*Inappropriate Prescribing*’ [4]. The use of a medication for which the associated risks outweigh the expected benefits can be considered as inappropriate, especially if an alternative treatment has been shown to be safer and more effective. According to a report published by the French National Authority for Health, both prescription of medication for excessively long periods and the failure to prescribe recommended medications can be classified as inappropriate prescribing [5]. In addition, the prescription of medications that have a high risk to interact with other drugs, or with the disease can also be considered as inappropriate. All of these examples will be herein described as inappropriate prescription (IP).

Many tools have been developed to detect IP in the elderly. This is largely due to the susceptibility of the elderly to disease and the prevalence of polypharmacy in this population. The *Beers Criteria for Potentially Inappropriate Medication Use in Older Adults* [6] were the first criteria to be proposed and are also the most well-known. However, one major disadvantage of this tool is that it includes many medications that are not sold in Europe. In 2008, Gallagher *et al.* developed a tool called STOPP/START (*Screening Tool of Older Person’s Prescriptions/Screening Tool to Alert doctors to Right Treatment*) that comprises two medication lists [7]. The ‘STOPP’ list includes prescriptions that should be stopped and the ‘START’ list includes prescriptions that should be initiated, in the absence of any contra-indication. This system is particularly useful because it classifies drugs according to various medical conditions that are commonly found in the elderly. In a study in 2008, the use of the STOPP list identified IPs in 35% of a cohort of elderly patients and one third of these IPs were associated with an adverse drug event [8]. Another study involving randomized hospitalized patients showed that the occurrence of IP was 35% lower in patients who were prescribed drugs according to STOPP/START criteria than in patients for who usual pharmaceutical criteria were used [9]. However, so far no tool has been created to the pediatric population.

Our objective was to create the first IP tool in pediatrics, which we called POPI (Pediatrics: Omission of Prescriptions and Inappropriate prescriptions) [10]. Our objective was to raise awareness about this tool and to validate its content through a network of medical professionals working in pediatrics.

## Materials and Methods

POPI should contain around 100 propositions that were classified according to biological system and classified according to whether they involve an omitted or an inappropriate prescription. The propositions were further divided within these two lists according to the major biological systems (as this was done for other geriatric tools [6], [8]). We decided to include around 100 propositions: this was a good compromise between the number of major biological systems to explore, the number of items in the geriatric lists and the maximum number of items compatible with a tool easy use.

This project began in the Robert-Debré University Hospital, AP-HP (Assistance Publique-Hôpitaux de Paris) in Paris, France. POPI is comprised of a list of health problems frequently encountered in pediatrics. These problems were chosen in 2010 according to the following criteria, as concerns pediatrics: their frequency in the general population, the reasons for hospitalization (listed in the French hospital system’s medico-administrative database in 2011 ‘programme de médicalisation des systèmes d’information’ [PMSI] at the Robert-Debré University Hospital), and their prevalence according to data from the French National Health Insurance Fund for Employees (la caisse nationale de l’assurance maladie des travailleurs salariés [CNAMTS]) of long-term illnesses [11]. According to these criteria, we selected health problems requiring either drug intervention, or no pharmacological intervention whatsoever (i.e. treatment in such cases would be considered as inappropriate).

For each disease, we considered the recommended pharmacological treatments, the risks of errors, contra-indications, drug-drug interactions, drug-disease interactions, and issues associated with dose and route.

For each of the chosen themes (or diseases), we established a literature search strategy to retrieve management recommendations. We selected only recommendations that were both backed up by evidence and were published after 2000. Recommendations were weighted according to their publication date. Data was obtained from learned or professional societies or agencies in France, the United States, or Great Britain: the French Health Products Safety Agency (ANSM or *Agence Française de Sécurité Sanitaire des Produits de Santé*), the French National Authority for Health (*Haute Autorité de Santé Française*), the French Society for Pediatricians (*Société Française de Pédiatrie*), the American Academy of Pediatrics (National Guideline Clearing House), and finally the National Institute for Health and Clinical Evidence, Cochrane Library (UK). We used the following databases for the origin of pharmacological agents, the commercially available forms, and potential drug-drug interactions: Thériaque [12], Micromedex [13], Lexi-Comp’s Pediatric & Neonatal Dosage Handbook [14], and the French medical journal ‘La Revue Prescrire’ [15]. We also used the MEDLINE database to search for examples of medication error and inappropriate prescription.

We validated the propositions included in POPI by a two round Delphi method [16], [17]. The aim of the Delphi method is to achieve a convergence of opinion and a general consensus on a particular topic, by questioning experts through successive questionnaires. The experts were chosen according to their area of expertise, and included pediatricians most of who are members of the French Society of Pediatricians, and pharmacists mostly members of the French Society of Clinical Pharmacy. Each expert has disclosed his conflicts of interest.

The fisrt round questionnaire comprised all of the propositions included in POPI draft, which were graded according to a nine-point Likert scale for agreement. A score of 1 indicates ‘total disagreement’ whereas a score of 9 ‘total agreement’, with intermediate values indicating degrees of agreement between these two extremes. The experts were also encouraged to make suggestions about the dose, the frequency, and the duration of treatment, provided that they could cite appropriate references to back up these suggestions. The experts could also comment on the propositions. The questionnaire was available online via the website ‘SurveyMonkey’, which is a tool designed to conduct web-based surveys [18].

Each of the panelists who had participated in the first round was sent the second-round questionnaire. These panelists were also given feedback on the results of the first round (their own previous individual ratings, median panel rating, and frequency distribution of the agreement rating). The panelists were then asked to re-rate each proposition based on both their own opinion and the group response to the previous round.

Only the propositions that obtained a median score in the upper tertile (between 7 and 9) with an agreement of more than 65% of participants in the first round of Delphi were retained. These propositions were modified according to the experts’ comments, and were subjected to a second round of questioning. Only the propositions that obtained a median score between 7 and 9 with an agreement of more than 75% of participants in this second round were retained. The experts had two weeks to reply to the questionnaire. For both the first and second questionnaire, a reminder was sent out one and two weeks before the deadline.

Experts characteristics were also noted, including their age, their place of work, and their number of years of experience.

Qualitative data are expressed as numbers (percentages) and quantitative data as median (quartiles) and minima, maxima. SAS software (VERSION 9.3) was used for statistical analysis.

The study was reviewed and approved by the Robert-Debré institutional review board.

## Results

The first draft of POPI contained 108 propositions: 80 propositions of Inappropriate Prescription (IP) and 28 propositions of Omission of Prescription (OP). These propositions were classified into five broad categories: digestive problems (n = 15); Ear, Nose and Throat (ENT) problems or pulmonary problems (n = 23); dermatological problems (n = 30); neuropsychiatric disorders (n = 16); and diverse illnesses (n = 24). Each category was further divided into several medical conditions. We contacted 33 experts between June and September 2012. Sixteen experts agreed to participate in the development of the POPI tool. The median expert age was 49 years, range [32–66 years] and their median number of years of experience was 25 years, range [3–40 years]. The ratio of pediatricians to pharmacists was 1∶1. Half were working in a hospital environment and the other half were working in the community. Each physician working within a hospital environment was specialized in a particular medical domain: endocrinology, hematology, nephrology, cancerology, or pulmonology.

**Figure 1:**
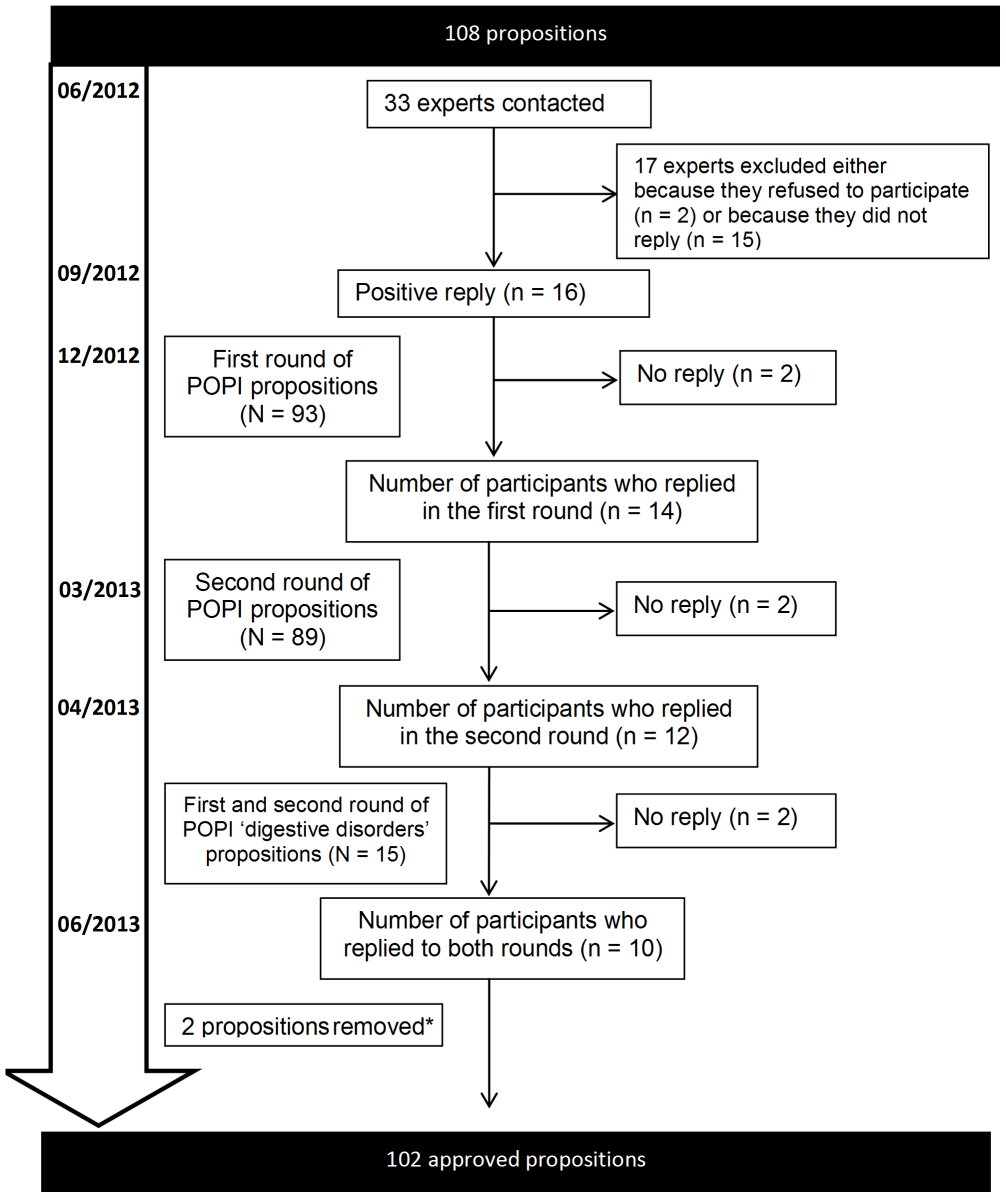
Workflow for the validation of POPI. *An item involving codeine was removed subsequent to the validation of the propositions included in POPI, following the revelation of new contraindications for this drug in children under 12 years old [22]. N: Number of items; n: number of panelists.

Figure 1 shows the workflow of the study. The first round questionnaire was sent to the 16 experts at the start of December 2012 and the replies were collected by the start of January 2013; 14 (14/16, 87.5%) participants responded to the first round of questions. Two propositions received 13 replies because one expert did not use the answer grid properly. More than 65% of the panelists gave top-tertile (7–9) agreement to 93 propositions. Ten propositions were modified according to the experts’ comments during this first round of questions producing 93 propositions for the second round.

The second questionnaire was submitted at the end of March 2013 and the replies were collected within one month. During this second round of questions, 85.5% (12/14) of participants replied. More than 75% of the panelists gave top-tertile agreement to all the 93 propositions submitted.

The propositions involving the category ‘digestive problems’ (n = 15) were submitted separately in April 2013 for 2 rounds rating. All of these propositions were unanimously accepted during two rounds of questions that took place between April and May 2013. Ten experts participated in these rounds of questions (i.e. 71.5% of the 14 experts who replied in the initial survey carried out between December 2012 and January 2013.

Table 1 shows the 102 propositions that were validated for use in POPI. A proposition involving codeine was removed subsequent to the validation of POPI, following the revelation of new contraindications for this drug in children under 12 years old [19]. Another proposition about the use of permethrin for lice was removed because of new recommendation to use dimeticone first (lack of resistance) [20]. Table 2 summarizes the references justification for each table 1 pathology.

**Table 1:** Propositions validated for use in POPI.

**DIVERSE** **ILLNESSES**	**PAIN AND FEVER**
	**Inappropriate prescriptions**
	Prescription of two alternating antipyretics as a first-line treatment
	Prescription of a medication other than paracetamol as a first line treatment (except in the case of migraine)
	Rectal administration of paracetamol as a first-line treatment
	The combined use of two NSAIDs
	Oral solutions of ibuprofen administered in more than three doses per day using a graduated pipette of 10mg/kg (other than Advil)
	Opiates to treat migraine attacks
	**Omissions**
	Failure to give sugar solution to new-born babies and infants under four months old two minutes prior to venipuncture
	Failure to give an osmotic laxative to patients being treated with morphine for a period of more than 48 hours
	**URINARY INFECTIONS**
	**Inappropriate prescriptions**
	Nitrofurantoin used as a prophylactic
	Nitrofurantoin used as a curative agent in children under six years of age, or indeed any other antibiotic if avoidable
	Antibiotic prophylaxis following an initial infection without complications (except in the case of uropathy)
	Antibiotic prophylaxis in the case of asymptomatic bacterial infection (except in the case of uropathy)
	**VITAMIN SUPPLEMENTS AND ANTIBIOTIC PROPHYLAXIS**
	**Inappropriate prescriptions**
	Fluoride supplements prior to six months of age
	**Omissions**
	Insufficient intake of vitamin D. Minimum vitamin D intake: Breastfed baby = 1 000 to 1 200 IU/day; Infant <18 months of age (milk enriched in vitamin D) = 600 to 800 IU/day; Child aged between 18 months and five years, and adolescents aged between 10 and 18 years: two quarterly loading doses of 80 000 to 100 000 IU/day in winter (adolescents can take this dose in one go)
	Antibiotic prophylaxis with phenoxymethylpenicillin (Oracilline) starting from two months of age and lasting until five years of age for children with sickle-cell anemia: 100 000 IU/kg/day (in two doses) for children weighing 10kg or less and 50 000 IU/kg/day for children weighing over 10kg (also in two doses)
	**MOSQUITOS**
	**Inappropriate prescriptions**
	The use of skin repellents in infants less than six months old and picardin in children less than 24 months old
	Citronella (lemon grass) oil (essential oil)
	Anti-insect bracelets to protect against mosquitos and ticks
	Ultrasonic pest control devices, vitamin B1, homeopathy, electric bug zappers, sticky tapes without insecticide
	**Omissions**
	** DEET:** “30%” (max) before 12 years old; “50%” (max) after 12 years old
	** IR3535**: “20%” (max) before 24 months old; “35%” (max) after 24 months old
	Mosquito nets and clothes treated with pyrethroids
**DIGESTIVE** **PROBLEMS**	**NAUSEA, VOMITTING, OR GASTROESOPHAGEAL REFLUX**
	**Inappropriate prescriptions**
	Metoclopramide
	Domperidone
	Oral administration of an intravenous proton pump inhibitor (notably by nasogastric tube)
	Gastric antisecretory drugs to treat gastroesophageal reflux, dyspepsia, the crying of new-born babies (in the absence of any other signs or symptoms), as well as faintness in infants
	The combined use of proton pump inhibitors and NSAIDs, for a short period of time, in patients without risk factors
	The use of type H2 antihistamines for long periods of treatment
	Erythromycin as a prokinetic agent
	The use of setrons (5-HT3 antagonists) for chemotherapy-associated nausea and vomiting
	**Omissions**
	Oral rehydration solution
	**DIARRHEA**
	**Inappropriate prescriptions**
	Loperamide before 3 years of age
	Loperamide in the case of invasive diarrhea
	The use of Diosmectite (Smecta) in combination with another medication
	The use of Saccharomyces boulardii (Ultralevure) in powder form, or in a capsule that has to be opened prior to ingestion, to treat patients with a central venous catheter or an immunodeficiency
	Intestinal antiseptics
	**Omissions**
	Oral rehydration solution
**–ENT-PULMONARY** **PROBLEMS**	**COUGH**
	**Inappropriate prescriptions**
	Pholcodine
	Mucolytic drugs, mucokinetic drugs, or helicidine before two years of age
	Alimemazine (Theralene), oxomemazine (Toplexil), promethazine (Phenergan, and other types)
	Terpene-based suppositories
	**Omissions**
	Failure to propose a whooping cough booster vaccine for adults who are likely to become parents in the coming months or years (only applicable if the previous vaccination was more than 10 years ago). This booster vaccination should also be proposed to the family and entourage of expectant parents (parents, grand-parents, nannies/child minders)
	**BRONCHIOLITIS IN INFANTS**
	**Inappropriate prescriptions**
	Beta2 agonists, corticosteroids to treat an infant’s first case of bronchiolitis
	H1-antagonists, cough suppressants, mucolytic drugs, or ribavirin to treat bronchiolitis
	Antibiotics in the absence of signs indicating a bacterial infection (acute otitis media, fever, etc.)
	**Omissions**
	0.9% NaCl to relieve nasal congestion (not applicable if nasal congestion is already being treated with 3% NaCl delivered by a nebulizer)
	Palivizumab in the following cases: (1) babies born both at less than 35 weeks of gestation and less than six months prior to the onset of a seasonal RSV epidemic; (2) children less than two years old who have received treatment for bronchopulmonary dysplasia in the past six months; (3) children less than two years old suffering from congenital heart disease with hemodynamic abnormalities
	**ENT INFECTIONS**
	**Inappropriate prescriptions**
	An antibiotic other than amoxicillin as a first-line treatment for acute otitis media, strep throat, or sinusitis (provided that the patient is not allergic to amoxicillin). An effective dose of amoxicillin for an pneumoncoccal infection is 80–90 mg/kg/day and an effective dose for a streptococcal infection is 50 mg/kg/day
	Antibiotic treatment for a sore throat, without a positive rapid diagnostic test result, in children less than three years old
	Antibiotics for nasopharyngitis, congestive otitis, sore throat before three years of age, or laryngitis; antibiotics as a first-line treatment for acute otitis media showing few symptoms, before two years of age
	Antibiotics to treat otitis media with effusion (OME), except in the case of hearing loss or if OME lasts for more than three months
	Corticosteroids to treat acute suppurative otitis media, nasopharyngitis, or strep throat
	Nasal or oral decongestant (oxymetazoline (Aturgyl), pseudoephedrine (Sudafed), naphazoline (Derinox), ephedrine (Rhinamide), tuaminoheptane (Rhinofluimicil), phenylephrine (Humoxal))
	H1-antagonists with sedative or atropine-like effects (pheniramine, chlorpheniramine), or camphor; inhalers, nasal sprays, or suppositories containing menthol (or any terpene derivatives) before 30 months of age
	Ethanolamine tenoate (Rhinotrophyl) and other nasal antiseptics
	Ear drops in the case of acute otitis media
	**Omissions**
	Doses in mg for drinkable (solutions of) amoxicillin or josamycin
	Paracetamol combined with antibiotic treatment for ear infections to relieve pain
	**ASTHMA**
	**Inappropriate prescriptions**
	Ketotifen and other H1-antagonists, sodium cromoglycate
	Cough suppressants
	**Omissions**
	Asthma inhaler appropriate for the child’s age
	Preventative treatment (inhaled corticosteroids) in the case of persistent asthma
**DERMATOLOGICAL** **PROBLEMS**	**ACNE VULGARIS**
	**Inappropriate prescriptions**
	Minocycline
	Isotretinoin in combination with a member of the tetracycline family of antibiotics
	The combined use of an oral and a local antibiotic
	Oral or local antibiotics as a monotherapy (not in combination with another drug)
	Cyproterone+ethinylestradiol (Diane 35) as a contraceptive to allow isotretinoin per os
	Androgenic progestins (levonorgestrel, norgestrel, norethisterone, lynestrenol, dienogest, contraceptive implants or vaginal rings)
	**Omissions**
	Contraception (provided with a logbook/diary) for menstruating girls taking isotretinoin
	Topical treatment (benzoyl peroxide, retinoids, or both) in combination with antibiotic therapy
	**SCABIES**
	**Inappropriate prescriptions**
	The application of benzyl benzoate (Ascabiol) for periods longer than eight hours for infants and 12 hours for children or for pregnant girls
	**Omissions**
	A second dose of ivermectin two weeks after the first
	Decontamination of household linen and clothes and treatment for other family members
	**LICE**
	**Inappropriate prescriptions**
	The use of aerosols for infants, children with asthma, or children showing asthma-like symptoms such as dyspnea
	**RINGWORM**
	**Inappropriate prescriptions**
	Treatment other than griseofulvin for Microsporum
	**Omissions**
	Topical treatment combined with an orally-administered treatment
	Griseofulvin taken during a meal containing a moderate amount of fat
	**IMPETIGO**
	**Inappropriate prescriptions**
	The combination of locally applied and orally administered antibiotic
	Fewer than two applications per day for topical antibiotics
	Any antibiotic other than mupirocin as a first-line treatment (except in cases of hypersensitivity to mupirocin)
	**HERPES SIMPLEX**
	**Inappropriate prescriptions**
	Topical agents containing corticosteroids
	Topical agents containing acyclovir before six years of age
	**Omissions**
	Paracetamol during an outbreak of herpes
	Orally administered acyclovir to treat primary herpetic gingivostomatitis
	**ATOPIC ECZEMA**
	**Inappropriate prescriptions**
	A strong dermocorticoid (clobetasol propionate 0.05% Dermoval, betamethasone dipropionate Diprosone) applied to the face, the armpits or groin, and the backside of babies or young children
	More than one application per day of a dermocorticoid, except in cases of severe lichenification
	Local or systemic antihistamine during the treatment of outbreaks
	Topically applied 0.03% tacrolimus before two years of age
	Topically applied 0.1% tacrolimus before 16 years of age
	Oral corticosteroids to treat outbreaks
**NEUROPSYCHIATRIC** **DISORDERS**	**EPILEPSY**
	**Inappropriate prescriptions**
	Carbamazepine, gabapentin, oxcarbazepine, phenytoin, pregabalin, tiagabine, or vigabatrin in the case of myoclonic epilepsy
	Carbamazepine, gabapentin, oxcarbazepine, phenytoin, pregabaline, tiagabine, or vigabatrin in the case of epilepsy with absence seizures (especially for childhood absence epilepsy or juvenile absence epilepsy)
	Levetiracetam, oxcarbamazepine in mL or in mg without systematically writing XX mg per Y mL
	**DEPRESSION**
	**Inappropriate prescriptions**
	An SSRI antidepressant other than fluoxetine as a first-line treatment (in the case of pharmacotherapy)
	Tricyclic antidepressants to treat depression
	**NOCTURNAL ENURESIS**
	**Inappropriate prescriptions**
	Desmopressin administered by a nasal spray
	Desmopressin in the case of daytime symptoms
	An anticholinergic agent used as a monotherapy in the absence of daytime symptoms
	Tricyclic agents in combination with anticholinergic agents
	Tricyclic agents as a first-line treatment
	**ANOREXIA**
	**Inappropriate prescriptions**
	Cyproheptadine (Periactin), clonidine
	**ATTENTION DEFICIT DISORDER WITH OR WITHOUT HYPERACTIVITY**
	**Inappropriate prescriptions**
	Pharmacological treatment before age six (before school), except in severe cases
	Antipsychotic drugs to treat attention deficit disorder without hyperactivity
	Slow release methylphenidate as two doses per day, rather than only one dose
	**Omissions**
	Recording a growth chart (height and weight) if the patient is taking methylphenidate

**Table 2:** References justification for each POPI statement.

**Pain and Fever**
Mise au point sur la prise en charge de la fièvre chez l’enfant – **AFSSAPS** –2005
Fever and Antipyretic use in children – American Academy of Pediatrics (AAP) –2011
Feverish illness in children – **NICE** –2007
Prise en charge médicamenteuse de la douleur aiguë et chronique chez l’enfant - **AFSSAPS** –2009
Prevention and Management of Pain in the Neonate - **AAP** –2006
**Urinary Infections**
Nitrofurantoïne et risque de survenue d’effets indésirables hépatiques et pulmonaires lors de traitements prolongés – **AFSSAPS** –2011
Urinary tract infection in children – **NICE** –2007
**Vitamin Supplements and Antibiotic Prophylaxis**
Utilisation du fluor dans la prévention de la carie dentaire avant l’âge de 18 ans – **AFSSAPS** –10/2008
Dents et fluor chez les enfants – **Idées-Forces Prescrire** – Novembre 2011
Alimentation du nourrisson et de l’enfant en bas âge. Réalisation pratique – **SFP** (Société Française de Pédiatrie) –2003
La Vitamine D : une vitamine toujours d’actualité chez l’enfant et l’adolescent. Mise au point par le Comité de nutrition de la Société française de pédiatrie – **SFP** –2012
Prise en charge de la drépanocytose chez l’enfant et l’adolescent – **HAS** –09/2005
**Mosquitos**
Protection Antivectorielle RBP – **Société Française de Parasitologie** –2010
**BEH** –29 mai 2012– n°20–21
Prévention des piqûres de moustiques ou des morsures de tiques – **Idées-Forces Prescrire** – Juin 2012
**Nausea, Vomitting, or Gastroesophageal Reflux**
Contre-indication des spécialités à base de métoclopramide (Primpéran et génériques) chez l’enfant et l’adolescent et renforcement des informations sur les risques neurologiques et cardiovasculaires – **AFSSAPS** - Lettre aux professionnels de santé –08/02/2012
Antisécrétoires gastriques chez l’enfant – **AFSSAPS** –06/2008
Pediatric Gastroesophageal Reflux Clinical Practice Guidelines – **NASPGHAN** –2009
Traitement médicamenteux des diarrhées aiguës infectieuses du nourrisson et de l’enfant - **SFP** –2002
Managing Acute Gastroenteritis Among Children: Oral Rehydration, Maintenance, and Nutritional Therapy - Centers for Disease Control and Prevention – **AAP** –2003
Diarrhoea and vomiting in children under 5– **NICE** –2009
**Diarrhea**
Diarrhoea and vomiting in children under 5– **NICE** –2009
Traitement médicamenteux des diarrhées aiguës infectieuses du nourrisson et de l’enfant – **SFP** –2002
Managing Acute Gastroenteritis Among Children: Oral Rehydration, Maintenance, and Nutritional Therapy - Centers for Disease Control and Prevention – **AAP** –2003
**Cough**
Pholcodine – **AFSSSAPS** –2011
Toux aiguë chez les enfants de moins de 2 ans – AFSSAPS –2010
**BHE** – Calendrier vaccinal –10 avril 2012– n°14–15
**Bronchiolotis in Infants**
Diagnosis and Management of Bronchiolitis – **AAP** –2006
Bronchiolite du nourrisson – Conférence de consensus – HAS –2000
Bronchiolite chez les nourrissons – Traitement – **Idées-Forces Prescrire –** Septembre 2011
**Ear Infections**
Antibiothérapie dans les infections respiratoires hautes – **SFP** –12/2011
Respiratory tract infections – **NICE** –2011
Rhume : traitements – **Idées-Forces Prescrire** – Avril 2011
Otite moyenne aiguë : traitement antibiotique – **Idées-Forces Prescrire** – Janvier 2011
Diagnosis and Management of Acute Otitis Media – **AAP** –2004
**Asthma**
**Global Initiative for Asthma** –2011
Asthme de l’enfant de moins de 36 mois : diagnostic, prise en charge et traitement en dehors des épisodes aigus – **HAS** – Mars 2009
Managing Asthma Long Term In Children 0–4 and 5–11 Years of Age – **NHLBI** –2007
**Acne Vulgaris**
Recommandations de bonne pratique – **AFSSAPS**–2007
Minocycline : restriction d’utilisation en raison d’un risque de syndromes d’hypersensibilité graves et d’atteintes auto-immunes – Lettre aux professionnels de santé – **ANSM** –2012
Isotrétinoïne orale – Renforcement du Programme de Prévention des Grossesses et rappel sur la survenue éventuelle de troubles psychiatriques – **AFSSAPS** –05/2009
**Scabies**
Sexually Transmitted Diseases Treatment Guidelines – **CDC** –2010
Gale – **Avis du conseil supérieur d’hygiène publique de France** –2003
**Lice**
Poux du cuir chevelu – **La Revue Prescrire N°365**–2014
**Ringworm**
Guidelines for the Management of Tinea Capitis in Children – **ESPD** –2010
**Impetigo**
Prescription des antibiotiques par voie locale dans les infections cutanées bactériennes primitives et secondaires – **AFSSAPS** –2004
**Herpes Simplex**
Prise en charge de l’herpès cutanéo-muqueux chez le sujet immunocompétent – **SFD** –2001
**Atopic Eczema**
Prise en charge de la dermatite atopique de l’enfant – **Société Française de Dermatologie** –2005
Atopic eczema in children – **NICE** –2007
Protopic – **HAS** – Commission transparence –2011
**Epilepsy**
Epilepsy – **NICE** –2012
Epilepsies graves – **HAS** –07/2007
**Depression**
Bon usage des antidépresseurs au cours de la dépression de l’enfant et de l’adolescent – **AFSSAPS** – Janvier 2008
Depression in children and young people – **NICE** –2009
**Nocturnal Enuresis**
Utilisation de la desmopressine (Minirin) dans l’énurésie nocturne isolée chez l’enfant – **AFSSAPS** –2006Nocturnal enuresis – **NICE** –2010
**Anorexia**
Anorexie : recommandation pour la pratique clinique – **HAS** – Juin 2010
**Attention Deficit Disorder with or withou Hyperactivity**
Attention deficit hyperactivity disorder Diagnosis and management of ADHD in children, young people and adults – **NICE** –2008
ADHD : Clinical Practice Guideline for the Diagnosis, Evaluation, and Treatment of Attention-Deficit/Hyperactiviy Disorder in Children and Adolescents – **AAP** –2010

## Discussion

POPI (Pediatrics: Omission of Prescriptions and Inappropriate prescriptions) is the first tool that has been designed to detect the omission of prescriptions or inappropriate prescriptions specifically in pediatric patients [21]. If polymedication is unusual for children, there are however multiple health care professional who prescribe or counsel drug for children: general practitioner, paediatricians, pharmacists, nurses, midwives etc.

The POPI criteria are based on the same classification system as the STOPP/START criteria, (i.e. according to the major biological systems [8]). We selected this form because such lists have been successfully used to detect preventable adverse drug events [8], [9], [22]. The Beers criteria were updated in 2012 to incorporate this classification system [6]. Our tool, which was developed using a Delphi method, was validated by 14 health care professionals. The Delphi method is one of the main method used for the development of tools designed to detect inappropriate prescriptions in geriatric patients [6], [8], [22]–[26]. The number of experts to develop geriatric tools vary between 11 and 32 and their specialties include pharmacy, psychopharmacology, pharmacology, pharmacoepidemiology, internal medicine or geriatrics [6], [7], [23], [24], [26]. For the validation of POPI, the number of experts in each category was equal so as to ensure that hospital and community environments were equally represented. There is currently no consensus regarding the composition of such panels of experts; there are no recommendations about the numbers or qualifications of experts to be included. More pharmacists were involved in the validation of the POPI criteria than in the validation of similar criteria that were developed for geriatrics. This strong representation is partly because the initial project was developed by hospital pharmacists. One limitation of our study in the absence of general practitioners from our panel of experts. Indeed, these doctors regularly deliver health care to children in the community and hence could greatly benefit from the use of POPI.

Few data about inappropriate prescriptions have been published in pediatric patients. Although studies have investigated medication errors [27]–[29], not one study has examined the link between the rate of medication errors and the rate of adverse drug events in pediatrics. In adults, it is estimated that around one adverse drug occurs for every 100 medication errors [30], [31]. There is increasing recognition that rational prescribing is an important issue in children [2].

The different propositions included in POPI were based on recommendations from recognized learned and academic societies and were preselected by the initial working group. Of the 108 propositions, 104 were validated by experts in the first round of Delphi, and all of the propositions submitted in the second round were subsequently validated. The final version of the POPI criteria contains 79 examples of inappropriate prescription and 25 examples of omission of prescription. The modifications that were made during the first and second rounds of Delphi involved refinements in the phrasing and exact details of the propositions. Overall, the experts were very responsive, and we collected around 80% of replies within three weeks of sending the questionnaires. The feedback of the experts was very positive and many of them commented that they were very interested in the development of POPI. The STOPP/START criteria contained as many propositions to validate as the POPI criteria. For STOPP/START, a consensus was obtained for 77 out of 80 propositions that were submitted in the first round [7]. For the criteria developed by Laroche *et al.* a consensus was reached for 33 out of 37 criteria during the first round [26]. This illustrates the importance of preselecting the propositions prior to their submission to experts, to ensure that a consensus will be reached on the largest possible number of propositions. The time that experts were given to reply to questionnaires during the development of criteria similar to POPI is often not stated, with the exception of STOPP/START, in which all answers were obtained within two months [7]. We estimated that one month (a minimum of two weeks with two reminders) was a reasonable amount of time for the completion of the questionnaire. This time constraint was applied to both rounds of questions.

Our criteria contain more propositions than the STOPP/START criteria (83 propositions vs. 102 for POPI) and more than the updated 2012 Beers criteria (85 propositions). The classification of these propositions by biological system makes the POPI criteria fast to use, and POPI considers only those medical conditions that require prescriptions. The categories that we used are not the same as those in the STOPP/START criteria or the updated 2012 Beers criteria because diseases that affect children are not the same as those that affect the elderly. Indeed, in most criteria designed for use in geriatrics, psychiatry and cardiology constitute major categories [6], [7], [26], whereas the categories that contain the most propositions in POPI are respiratory problems, gastroenterology, and dermatology.

The POPI criteria have not yet been tested in the setting of routine prescriptions and needs validating clinically. Two studies will be carried out with this objective in mind. One study will examine the degree of inter-rater agreement of the various propositions of POPI, by assessing the percentage of concordance corrected for chance agreement, termed κ (Kappa). This will provide a measure of the precision of the POPI criteria. A second study will examine the capacity of the POPI criteria to identify medication errors and evaluate the safety of drug used (involved drugs, indication) prospectively.

## Conclusion

We created the first set of criteria for the detection of inappropriate prescriptions and the omission of prescriptions in pediatrics. The resulting tool, named POPI, is available to all medical professionals (clinicians, pharmacists, in hospital or community working environment) liable to prescribe or dispense medication to children.
