# Correction: POPI (Pediatrics: Omission of Prescriptions and Inappropriate Prescriptions): Development of a Tool to Identify Inappropriate Prescribing

**DOI:** 10.1371/journal.pone.0108007

**Published:** 2014-09-16

**Authors:** The PLOS ONE Staff

There are errors in the legend for Figure 1, “Workflow for the validation of POPI.” The complete, correct legend for Figure 1 is:

*An item involving codeine was removed subsequent to the validation of the propositions included in POPI, following the revelation of new contraindications for this drug in children under 12 years old [19]. Another item involving permethrin for lice was removed due to new recommendations to use dimeticone first instead of insecticides [20]. N: Number of items; n: number of panelists
